# 

**Published:** 1949-04

**Authors:** 


### CONTENTS

| | Page |
|---|---|
| **Early Life.** By A. V. NEALE, M.D., F.R.C.P. .. .. .. | 31 |
| **Arteriography of the Lower Limbs.** By H. D. MOORE., F.R.C.S. | 37 |
| **The Surgery of Vascular Disease.** By R. MILNES WALKER, M.S., F.R.C.S. .. .. .. .. .. .. .. .. .. | 42 |
| **The Second Carey Coombs Memorial Lecture. "The Radiology of Rheumatic Heart Disease."** By SIR JOHN PARKINSON, M.D., F.R.C.P. | 46 |
| **Psychogenic Rheumatism: Its Differentiation from True Rheumatic Disease.** By PHILIP S. HENCH, M.D. .. .. .. | 48 |
| **Editorial Notes** .. .. .. .. .. .. .. .. | 51 |
| **Editor's Report** .. .. .. .. .. .. .. .. | 51 |
| **Obituary** .. .. .. .. .. .. .. .. .. | 55 |
| **Reviews of Books** .. .. .. .. .. .. .. .. | 55 |
| **Meetings of Societies** .. .. .. .. .. .. .. | 58 |
| **Local Medical Notes** .. .. .. .. .. .. .. | 61 |

